# Histone lactylation: from tumor lactate metabolism to epigenetic regulation

**DOI:** 10.7150/ijbs.91492

**Published:** 2024-03-03

**Authors:** Xiaoning Yu, Jing Yang, Jin Xu, Haoqi Pan, Wei Wang, Xianjun Yu, Si Shi

**Affiliations:** 1 Department of Pancreatic Surgery, Fudan University Shanghai Cancer Center, Shanghai, People's Republic of China.; 2 Department of Oncology, Shanghai Medical College, Fudan University, Shanghai, People's Republic of China.; 3 Shanghai Pancreatic Cancer Institute, Shanghai, People's Republic of China.; 4 Pancreatic Cancer Institute, Fudan University, Shanghai, People's Republic of China.; 5 Department of Pancreatic Surgery, Fudan University Shanghai Cancer Center, No.270 Dong' An Road, Shanghai, People's Republic of China.; 6 Department of Oncology, Shanghai Medical College, Fudan University, Shanghai, People's Republic of China.; 7 Shanghai Pancreatic Cancer Institute, No.270 Dong' An Road, 200032, Shanghai, People's Republic of China.; 8 Pancreatic Cancer Institute, Fudan University, Shanghai, People's Republic of China.; 9 Department of Pancreatic Surgery, Fudan University Shanghai Cancer Center, No.270 Dong' An Road, Shanghai, People's Republic of China.; 10 Department of Oncology, Shanghai Medical College, Fudan University, Shanghai, People's Republic of China.; 11 Shanghai Pancreatic Cancer Institute, No.270 Dong' An Road, 200032, Shanghai, People's Republic of China.; 12 Pancreatic Cancer Institute, Fudan University, Shanghai, People's Republic of China.

**Keywords:** Warburg Effect, Lactate, Histone lactylation, Tumor microenvironment

## Abstract

The Warburg Effect is one of the most well-known cancer hallmarks. This metabolic pattern centered on lactate has extremely complex effects on various aspects of tumor microenvironment, including metabolic remodeling, immune suppression, cancer cell migration, and drug resistance development. Based on accumulating evidence, metabolites are likely to participate in the regulation of biological processes in the microenvironment and to form a feedback loop. Therefore, further revealing the key mechanism of lactate-mediated oncological effects is a reasonable scientific idea. The discovery and refinement of histone lactylation in recent years has laid a firm foundation for the above idea. Histone lactylation is a post-translational modification that occurs at lysine sites on histones. Specific enzymes, known as “writers” and “erasers”, catalyze the addition or removal, respectively, of lactacyl group at target lysine sites. An increasing number of investigations have reported this modification as key to multiple cellular procedures. In this review, we discuss the close connection between histone lactylation and a series of biological processes in the tumor microenvironment, including tumorigenesis, immune infiltration, and energy metabolism. Finally, this review provides insightful perspectives, identifying promising avenues for further exploration and potential clinical application in this field of research.

## 1. Introduction

Lactate, a molecule ubiquitous in its familiarity, emerges as a frequent byproduct of the glycolytic pathway. Within the intricate landscape of glucose's oxidative metabolism, pyruvate assumes a pivotal role, orchestrated by the enzymatic action of lactate dehydrogenase (LDH), leading to the formation of L-lactate (herein referred to as "lactate," unless specified otherwise) 1. The reaction described above, called glycolysis, is a common energy-fueling reaction that occurs widely in mammalian cells 2. Historically, lactate has been thought of as a marginal metabolic waste product. It was not until the 1920s that Otto Warburg's findings illuminated the phenomenon that tumor cells exhibit aberrant glycolytic behavior, leading to the excessive production of lactate even in the presence of ample oxygen 3. In recent decades, propelled by the ongoing advancements in oncology, the importance of lactate within tumors has received increasing recognition, challenging its conventional classification as a metabolic waste. Lactate intricately regulates multiple intracellular and extracellular dynamics, including gene expression, metabolic dynamics, the tumor microenvironment (TME), and even the activation or inactivation of immune cells 4, 5.

Histones are indispensable constituents of chromosomes. Double-stranded DNA partially wraps around histones, spanning approximately 147 base pairs per coil. These intricate DNA-protein assemblies are termed nucleosomes, and each nucleosome is comprised of a quartet of histones: H2A, H2B, H3, and H4 6. Histones do not maintain a static presence within the chromatin; conversely, spurred by Vincent Allfrey's groundbreaking revelations in the 1960s, it became evident that lysine residues in proximity to the N-terminus of histones are susceptible to dynamic modifications. These alterations cover a spectrum of changes, including acetylation, methylation, and phosphorylation 7. They are collectively denoted histone posttranslational modifications (HPTMs). In 1997, a high-resolution X-ray analysis of nucleosome structure revealed the capacity of these HPTMs to impact chromatin architecture 8. Subsequently, as research efforts have intensified, the understanding of HPTM mechanisms has evolved into a more comprehensive framework.

As research has advanced, scholars have revealed a discernible pattern wherein intra- and extracellular metabolic processes significantly affect gene expression, thereby facilitating the establishment of a pervasive and rational feedback regulatory mechanism. Various HPTMs, as exemplified by acetylation, serve as elucidative examples of this phenomenon. Consequently, a plausible conjecture emerged. Lactate, a preeminent and prevalent metabolic byproduct, is exceedingly likely to engage in multiple processes that affect HPTMs, culminating in a diverse spectrum of downstream effects. Despite the omnipresence of lactate, its potential histone modification modalities and multiple prospective roles have been conspicuously neglected in previous research, inviting further investigation by the scientific community. A pivotal advancement occurred in 2019 when Zhang and colleagues first published their research revealing a novel HPTM: histone lactylation 9. Zhang also reported that this epigenetic regulation is associated with macrophage polarization, which possibly links histone lactylation with clinical conditions, including different kinds of cancer and inflammatory diseases 9. Subsequently, many scholars have continued to perform further studies to further elucidate the mechanism of histone lactylation and discover the concrete roles of this HPTM modality in many different kinds of diseases 1, 10.

To provide a more comprehensive exposition of the impact of histone lactylation on cancer, the authors have meticulously reviewed this topic. Commencing from the inception of lactate production, we present a holistic landscape of the dynamic shifts in lactate levels within tumor tissues, encompassing its generation, transport, consumption, and diverse functional roles. Subsequently, this review focuses on histone lactylation, delivering a systematic recapitulation of its effects. This review contributes to a deeper understanding of histone lactylation and offers novel insights into the regulation of tumor cell metabolism and the TME by lactylation. Moreover, drawing upon this cohesive framework, we offer an outlook on potential trajectories for future research advancements and plausible clinical applications.

## 2. The Warburg Effect and tumor lactate metabolism

The identification of the Warburg effect dates back almost a century; however, it took nearly a dozen years for the importance of this metabolic paradigm to be elucidated. Indeed, this mode of metabolism has a comprehensive influence extending beyond tumor cell proliferation to encompass the TME, thereby shaping an immunosuppressive milieu. Within this section, the authors present and discuss the role of lactate metabolism in cancer.

### 2.1 The Warburg Effect: A classical finding

Aligned with contemporary advancements in oncological research, the extensive identification and acknowledgment of metabolic reprogramming in tumor cells have solidified into a widely shared consensus among scholars within the field. Central to the discourse on tumor metabolism stands Otto Warburg, whose seminal discoveries have laid the cornerstone for subsequent investigations into this intricate process. During the 1920s, Warburg astutely revealed that tumor cells overwhelmingly favored the energy metabolism pathway of glycolysis 3, 11. In 1929, another pioneer in tumor metabolomics, Herbert Crabtree, revealed that tumor cells may have the ability to regulate oxidative phosphorylation (OXPHOS) reactions 12. Moreover, scholars have proposed a series of hypotheses regarding the development of cancer, although there have been many mistakes and limitations 13. The above findings, after being collated by Efraim, were the first to appear in publications under the name "Warburg effect", then a series of novel hypothesis regarding tumorigenesis emerged, positing that disrupted growth factor regulation precipitates aberrant cellular metabolism. This metabolic shift, toward glycolysis triggers the intricate process of tumorigenesis 2, 14, 15. Amidst the diverse novel facets of cancer, glycolytic metabolism and the Warburg effect continue to command sustained attention and act as an important focal point in tumor biology.

### 2.2 Lactate metabolism in tumor cells: Expansion of the Warburg Effect

**Figure 1:**
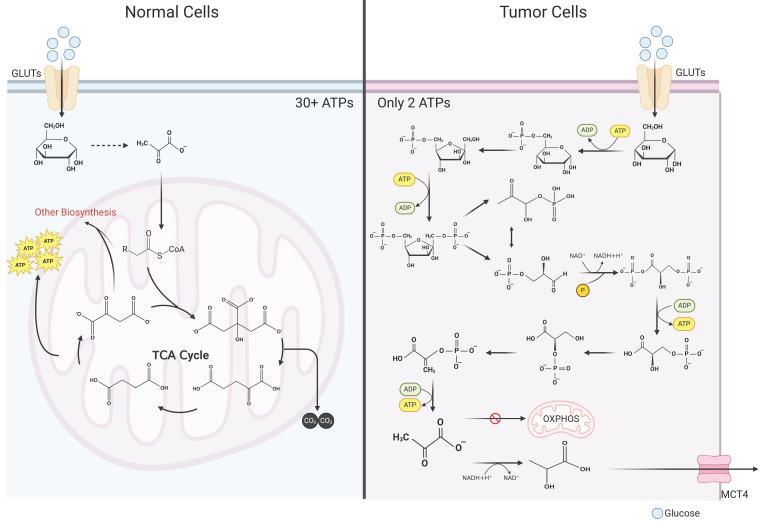
Comparison of glycolysis and oxidative phosphorylation. Contrasting glucometabolic of tumor cells and normal cells. In tumor cells, after glucose enters the cytosol, it undergoes a series of reactions to generate pyruvate. Pyruvate in a large proportion of tumor cells does not enter mitochondria for oxidative phosphorylation, but instead utilizes the reduced hydrogen atom provided by NADH in the cytosol to generate lactate. Since glucose is not completely oxidized, only 2 molecules of ATP can be generated after one molecule of glucose undergoes glycolytic metabolism. In normal cells, glucose is first oxidized to pyruvate in the cytosol, which is a process that bears great similarity to glycolytic metabolism in tumor cells. Subsequently, pyruvate is converted into the form of acetyl-CoA to enter the mitochondria where it reacts with oxaloacetate to generate citrate, which in turn enters the tricarboxylic acid cycle. Within the tricarboxylic acid cycle, acetyl-CoA is exhaustively oxidized to CO_2_ and generates NADH and FADH_2_, and subsequently these reduced hydrogen atoms are exhaustively oxidized in the electron transport chain and give rise to a large number of ATP molecules (more than 30 ATP molecules) that supply the cell energy (Created with BioRender.com). Abbreviations: GLUT, glucose transporter; MCT, monocarboxylate transporter; OXPHOS, oxidative phosphorylation; TCA, tricarboxylic acid cycle.

It is widely acknowledged that glycolytic metabolism exhibits significantly lower energy output efficiency in comparison to OXPHOS 16. In typical cellular contexts, glucose undergoes initial oxidation to pyruvate within the cytosol, followed by entry into the mitochondria, where it enters the tricarboxylic acid (TCA) cycle. Given that glucose is entirely oxidized to carbon dioxide, OXPHOS maximizes ATP generation, obviating lactate production 17. Oxidation of one glucose molecule can produce more than 36 molecules of ATP (depending on the metabolic pathway) 17. Only 2 ATP molecules can be produced in glycolytic metabolism (Figure 1) 18. Why do tumor cells preferentially use such an inefficient metabolic pathway? For a long time, the answer to that question was unclear. As the understanding of cancer continues to advance, the mystery of tumor lactate metabolism has gradually unraveled. In the ensuing sections, the authors meticulously discuss tumor cell lactate metabolism across four distinct aspects: production, transportation, utilization, and signal transduction.

#### 2.2.1 The production of lactate in tumor cells

The utilization of glucose by cells and the subsequent generation of lactate through glycolysis constitute pivotal metabolic events inherent to mammalian cells. For example, during instances of physical exertion, when the consumption of ATP molecules surpasses the energy production of glucose OXPHOS, glycolysis is invoked to supplement the energy supply, thus mitigating the relative insufficiency in ATP provisioning. Due to its streamlined reaction steps, glycolysis quickly yields a substantial quantity of ATP molecules, despite its inherent inefficiency 19. Lactate accumulation occurs as a result; serum lactate concentrations can reach 15 mmol/L during intense exercise compared to only 3 mmol/L in the resting state 19. The role of lactate in the development and progression of human diseases, such as cancer, infections, and inflammatory diseases, has also been studied to some extent 20. Especially in the TME, the lactate concentration can reach 40 mmol/L in the core region and more than 30 mmol/L in other locations 21. Active glycolytic metabolism in tumor cells may achieve a faster proliferation rate to meet their own energy needs 22. Changes in metabolic modalities such as those described above rely on alterations in all upstream regulatory molecules, downstream metabolites, and metabolic enzymes as intermediates, ultimately forming a feedback loop 23. New research indicates that tumor cells can exhibit metabolic features of both glycolysis and OXPHOS, and this process depends on the location of the tumor cells 24. Tumor cells located at a distance from microvessels typically exhibit heightened glycolytic activity, in contrast to their counterparts in close proximity to these vessels, which lean toward OXPHOS for high-efficiency energy acquisition. However, owing to the significantly accelerated growth rate of tumor cells relative to that of microvessels, glycolytic metabolism prevails on a macroscopic scale, leading to the overall production and accumulation of lactate. This lactate accumulation in the TME can surge to as high as 50 mmol/L 25. Another important source of lactate in tumor cells is glutamine, which first enters the cytosol via the type 2 amino acid transporter (ASCT2) and the sodium-coupled neutral amino acid transporter (SNAT2) under the regulation of c-Myc, a well-known proto-oncogene 26. Subsequently, under the catalysis of glutaminase and glutamate dehydrogenase, α-ketoglutaric acid (α-KG) is generated and enters the TCA cycle until pyruvate is generated, where it ultimately exits the cycle and produces lactate and ATP 26, 27. Another study first reported a novel lactate-producing pathway in pancreatic ductal adenocarcinoma (PDAC) cells, which suggested that metabolic substrates inside mitochondria can also enter the cytosol to produce lactate and ATP 28. Therefore, glutamate and glutamine are pivotal lactate-producing substrates that serve as molecular scaffolds and contribute essential carbon atoms. These pathways represent the most important secondary routes for lactate generation within tumor cells 20.

#### 2.2.2 The transport of lactate in tumors

With the deeper understanding of lactate metabolism, a comprehensive understanding of the lactate transport process has emerged. Previous studies have reported that exogenous lactate is readily accessible for cellular uptake and, intriguingly, that cells possess the capacity to intelligently modulate lactate uptake according to their requirements. Lactate is transported both intracellularly and intercellularly, with monocarboxylate transporters (MCTs) assuming the role of pivotal facilitators. The orchestration of these transport processes is mediated by factors such as pH, lactate concentration gradients, and cellular redox reactions 29. MCTs are a class of transmembrane transporters within the solute carrier family (SLC16), and 14 MCTs have been identified thus far 30, 31. Unfortunately, there is currently no X-ray crystallography analysis that successfully revealed the structure of MCTs, but scholars have obtained a basic understanding of these structures by means of functional simulation 31. The structure of MCTs includes 12 transmembrane helices labeled TM1-TM12. A semicircular structure in the middle of TM6 and TM7 was found to be intracellular, along with the N- and C-termini of the entire molecule 32-34. It is imperative to emphasize that the anchoring of MCTs to the cell membrane is contingent upon their interaction and cross-linking with the carboxyl terminus of the CD147 molecule 35. Therefore, certain scholars have referred to CD147, MCTs, and intracellular LDH, as the "lactate oxidation complex" 36. Lactate transportation is stimulated by the transmembrane concentration gradient, with each MCT having a determined direction of transportation, either into or out of the cell 37. Cells characterized by elevated intracellular lactate concentrations, thereby featuring upregulated surface MCT4 expression, facilitate the efflux of lactate from the cell. Conversely, cells harboring lower lactate concentrations primarily depend on MCT1 to mediate the uptake of lactate into the cytosol. This orchestrated group of MCTs underscores how distinct MCTs collaboratively construct a cohesive framework for the internal shuttling of lactate within tumors, thus preserving lactate homeostasis 30. The process of lactate transport by MCTs has been described in another study, so this topic will not be further discussed here 20. The relationship between MCTs and tumor progression is being revealed by an increasing number of studies because of the correlations between lactate levels and cancer patient outcomes. Thyroid stimulating hormone has been found to significantly increase MCT1 gene and protein expression in different tumors 37, 38. Other studies have reported that the upregulated expression of MCTs in tumor cells can be the result of the regulation of different signaling pathways, such as the hypoxia inducible factor (HIF) pathway, Myc pathway, Wnt pathway, and NF-κB pathway 39-43. Nevertheless, it has also been reported that hypermethylated MCT promoter regions within breast cancer cells are directly responsible for the heterogeneity of tumor cell metabolism 44. Overall, the pivotal role of MCTs is indispensable for facilitating the intercellular transport of lactate, thereby maintaining lactate homeostasis within the TME. Moreover, an in-depth exploration of the intricate interplay between MCTs and the trajectory of tumorigenesis holds promise and is imperative for advancing our comprehension of these mechanisms.

#### 2.2.3 The consumption of lactate in tumors

Macroscopically, the human body can indeed clear lactate via a metabolic pathway also known as the Cori cycle, in which lactate undergoes gluconeogenic metabolism as a raw material for the body to produce glucose 45. Lactate produced by peripheral tissues is metabolized within the liver and kidney, with acetyl-CoA serving as a raw material; this process eventually generates glucose, which can be transported to peripheral tissues or stored in the liver. From a microscopic perspective, delivering so much lactate to another distant organ is obviously inappropriate and impossible for tumors. In fact, tumors can take full advantage of the glucometabolic heterogeneity of different cells. Lactate is shuttled from cells undergoing glycolysis to those undergoing OXPHOS and can then be oxidized exhaustively 46. Another study reported related findings from another perspective, stating that the expression level of HIF may be negatively correlated with cellular OXPHOS 47. This observation suggested the potential for tumor cells of diverse metabolic phenotypes to congregate into a lactate metabolic network facilitated by MCT-mediated lactate transportation. Additionally, the LDH activity and lactate metabolic pathway scores likely mirror the proportion of cells within the tumor engaged in glycolytic versus oxidative phosphorylation processes 48, 49. This revelation introduces a novel perspective—namely, the conceptualization of tumor tissue as a metabolic community—promoting the consideration that the comprehensive realm of tumor metabolism might be perceived as an integrated entity.

#### 2.2.4 Lactate and signal transduction in tumors

**Figure 2:**
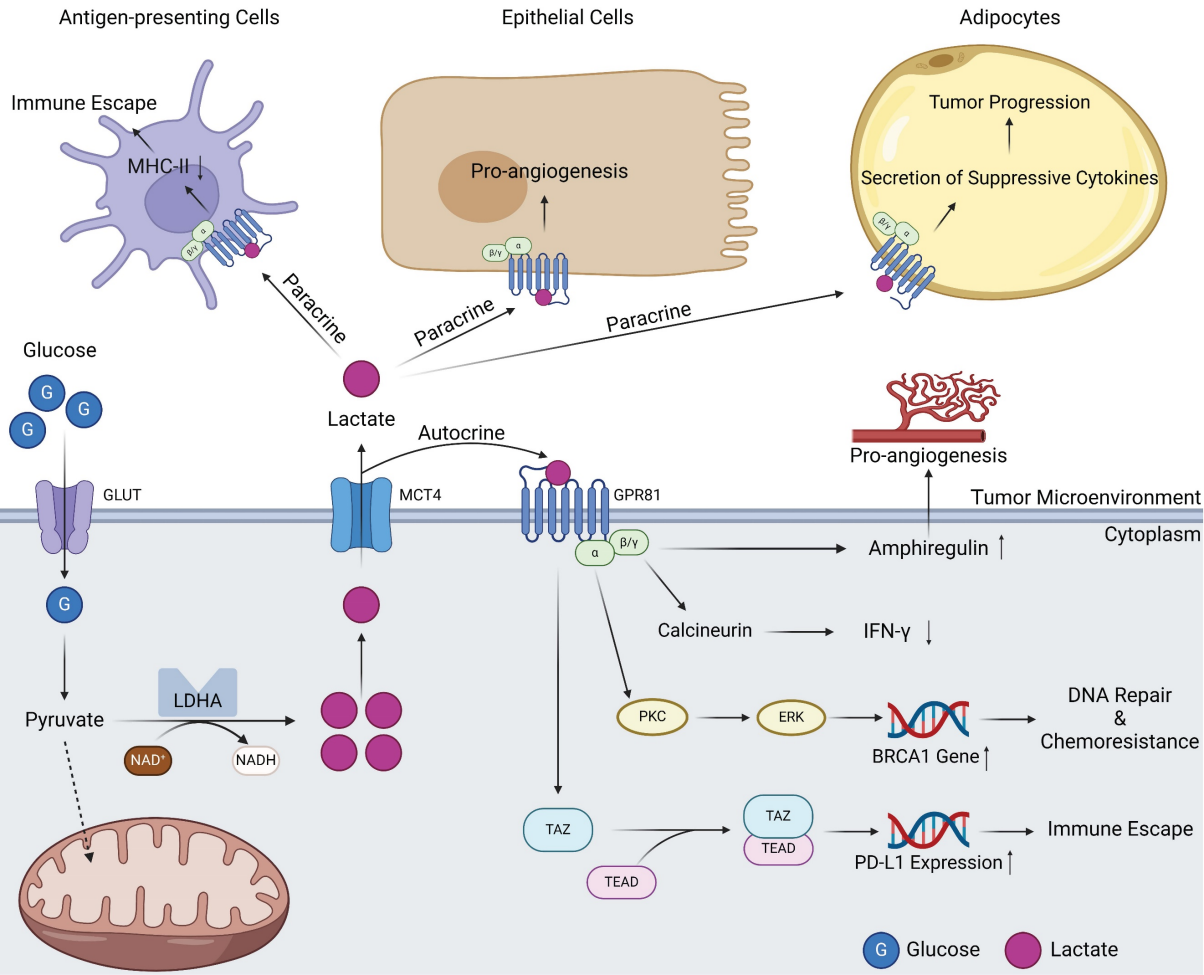
Signal transduction of GPR81 in tumor tissues. Lactate produced by intracellular metabolism can be transported into the TME by MCTs. On the one hand, these lactate molecules can act on the GPR81 receptor on the surface of tumor cells through autocrine effects. Subsequently, GPR81 transduced signals can mediate angiogenesis and IFN-γ reduction through the actions of amphiregulin and calcineurin. Signals derived from lactate can also activate BRCA1 expression through PKC\ERK pathway, thus promoting tumor progression and drug resistance. In addition, GPR81 can also directly activate the downstream TAZ/TEAD pathway, express immune checkpoint molecule PD-L1, promoting tumor immune escape. On the other hand, lactate in the TME can bind to GPR81 on the surface of other cells through paracrine effects, resulting in different effects on antigen-presenting cells, epithelial cells as well as adipocytes (Created with BioRender.com). Abbreviations: GLUT, glucose transporter; MCT, monocarboxylate transporter; LDHA, lactic dehydrogenase A; IFN-γ, interferon-γ.

The potential of lactate as a signaling molecule was largely overlooked until 2008, when a seminal report deepened the understanding of lactate-mediated signal transduction. Ge and colleagues pioneered the identification of GPR81, a G protein-coupled receptor (GPCR) capable of binding to lactate and relaying information intracellularly 50. When GPR81 is bound by lactate, the α-subunits immediately undergo structural conversion with energy generated by the GTP-GDP switch, which in turn affects downstream signaling molecules such as cAMP and Ca^2+^ 51. GPR81 inhibits the activation of protein kinase A system through decreasing the cAMP concentration 52. In normal human tissues, GPR81 is more highly expressed in adipose tissue, where it binds lactate, regulates inter- and intracellular transport, and, more importantly, inhibits lipolysis when intracellular lactate concentrations are excessive 53, 54. As research has progressed, the identification of multiple lactate receptors has emerged, with GPR132 playing a prominent role. However, the current research landscape remains circumscribed. A fascinating study revealed that the expression of GPR132 in macrophages may point to its potential involvement in orchestrating reactions within the TME 55, 56. Because of the close association between tumors and lactate levels, the relationship between GPR81 and tumors has also attracted attention, and in 2014, this relationship was first reported 44. High expression of GPR81 has been reported in PDAC and breast cancer cells. It is associated with tumor growth, invasion, TME immunosuppression, and T-cell anergy 24, 44, 57. The tumor-promoting role of GPR81 is related to its diverse signal transduction effects. On the one hand, GPR81 can exert effects on tumor cells in an autocrine-dependent manner (Figure 2). The first example is that the upregulation of GPR81 can reduce expression of interferon (IFN)-γ through the Gβγ-Ca^2+^-calcineurin pathway 58. Another example is associated with the well-known BRCA1 gene. GPR81 can elevate BRCA1 gene expression within tumor cells through the PKC-ERK pathway to aid tumor cells in DNA repair and ultimately lead to tumor chemotherapy resistance 59. A third example is related to immune checkpoints; GPR81 can upregulate PD-L1 expression through the TAZ/TEAD pathway to improve the immune evasion ability of tumor cells 60. On the other hand, GPR81 can influence other cells in the TME through paracrine effects (Figure 2) 61. Within the TME, lactate assumes functional roles through its interaction with GPR81 on various cell types, including epithelial cells, antigen-presenting cells (APCs), and adipocytes. These interactions manifest as proangiogenic effects, immune evasion mechanisms, and chemoresistance capabilities, respectively 62. In summary, GPR81 and other lactate receptors, represent a compelling subject of ongoing research. It is widely believed that the functions of these receptors within tumors extend beyond the scope outlined above, but additional receptors have yet to be identified, and a myriad of underlying mechanisms await elucidation. Ultimately, these endeavors hold potential for clinical translation in the future.

### 2.3 Research advance on lactate: Constructor of the suppressive TME

With the continuation of oncological research, the concept of the TME has becoming increasingly relevant 63. In 2014, a report revealed the role of lactate in tumor-associated macrophages (TAMs) 64. TAMs are composed of two different clusters, M1 and M2, which play antitumor and protumor roles, respectively 65. It was previously thought that macrophage polarization was triggered by upregulated HIF-1a and PPARγ coactivator-1a (PGC-1α) or by the pro-tumoral cytokines interleukin (IL)-4 and IL-13 66, 67. However, lactate has been recently found to recruit peripheral blood macrophages to infiltrate tumor sites in a dose-dependent manner and subsequently affect polarization by inducing the expression of vascular endothelial growth factor (VEGF) and arginase 1 (ARG1) 64. Since then, researchers have initiated a well-documented study of lactate levels and the TME, which has yielded many results (Table 1).

**Figure 3:**
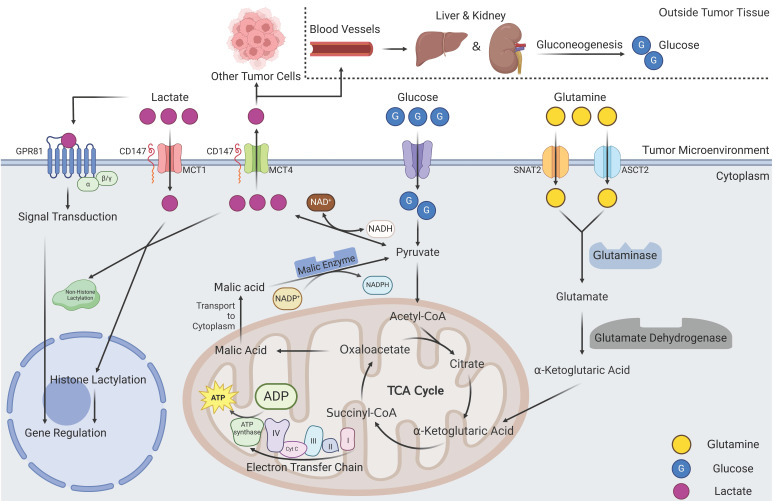
Overview of lactate metabolism in tumor tissues. Lactate production mainly comes from two pathways, one is glucose can directly generate lactate via pyruvate. Another pathway is that glutamine can be metabolized to α-ketoglutarate after entering the cell. α-ketoglutarate enters the mitochondria and participates in the tricarboxylic acid cycle, which, when the cycle proceeds to oxaloacetate, can exit the cycle in the form of malate and is catalyzed by malic enzyme to produce pyruvate and ultimately lactate. Intracellular lactate and lactate in the TME can undergo rapid transport by MCTs, a portion of lactate in the microenvironmental can enter the blood system to undergo gluconeogenic metabolism within the liver and kidney and regenerate glucose. Lactate in the microenvironment can also play signal transduction roles via GPR81 to influence gene expression of tumor cells. Intracellular lactate can participate in histone lactic acid modification, affecting cellular gene expression in an epigenetically regulated manner (Created with BioRender.com). Abbreviations: MCT, monocarboxylate transporter; SNAT2, sodium dependent neutral amino acid transporter; ASCT2, amino-acid transporter.

As the trajectory of oncology research has steadily advanced, an increasing number of inquiries concerning the Warburg effect and tumor lactate metabolism have been elucidated. However, concurrently, a plethora of questions remain to be explored, and numerous hypotheses await empirical validation. Indeed, it is imperative to refrain from construing the Warburg effect solely as an energy supply mechanism; rather, it is justifiable to assert that the centrality of the Warburg effect and lactate to tumor metabolism is far from an overstatement (Figure 3).

## 3. Histone lactylation: Linking lactate metabolism to epigenetic modifications

In the preceding discourse, we meticulously reviewed the pivotal importance of the Warburg effect within the context of tumors. Excessive lactate production affects metabolism, the biological behavior of cells, and the constitution of the TME, thereby establishing an intricate network. Nevertheless, while the broader importance of lactate in tumor biology has been substantially revealed, the specific molecular mechanisms underpinning its functions remain predominantly enigmatic. How precisely does lactate exert its regulatory influence? How intricately is lactate interwoven within the milieu of gene expression? Which specific genetic elements and metabolic pathways undergo alterations in response? These inquiries are worthy of elucidation. Importantly, in 2019, Zhang et al. published important research that contribute to the comprehension of these complex relationships 9. Subsequently, the landscape of research was invigorated by the emergence of histone lactylation as a novel focal point, and new information was gathered and reported. These achievements, marked by successive large-scale publications, have catalyzed groundbreaking progress across several domains, most notably within the field of oncology.

### 3.1 Discovery and refinement of histone lactylation

In 2019, an article by Zhang et al. reported a novel HPTM in histones related to lactate and partially clarified the upstream and downstream regulatory mechanism, namely, histone lactylation 9. As previously elucidated by the authors, the regulatory role of metabolites in gene expression serves as a ubiquitous mechanism that promotes cellular and systemic equilibrium. In essence, such regulation operates as a negative feedback loop influencing gene expression. Grounded in this prevalent understanding, Zhang and colleagues postulated that lactate might similarly participate in histone modification processes. This possibility was confirmed by observing a 72D mass shift at lysine sites on histones in MCF-7 cells. This initial observation served as a foundation, and fortunately, their hypothesis was further substantiated. Subsequent experimental endeavors revealed the occurrence of histone lactylation. Specifically, 26 lysine residues within the MCF-7 cell line and 16 within mouse bone marrow-derived macrophages (BMDMs) were identified as plausible candidates for this process 9. Following the discovery of this particular mode of HPTM, researchers have extensively researched it. Their pursuit extended beyond the modification itself, prompting contemplation about the origins of lactate molecules and the cascading downstream implications that this modification might herald. These factors require consideration. To further explore this phenomenon, 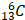 labeling was used to trace the trajectory of lactate. Remarkably, their investigations revealed that both intra- and extracellular lactate can stimulate lysine site lactylation (Kla). Notably, this stimulatory effect is contingent upon the lactate concentration, thereby introducing a concentration-dependent effect. Notably, intracellular lactate levels are intricately governed by the equilibrium between glycolysis and OXPHOS 9. However, the downstream mechanism of Kla is complicated by intricacies surpassing the relative simplicity of its upstream counterpart. Thus, by examining the polarization of macrophages as an example, Zhang and colleagues explored the function of Kla within this context. Their inquiry focused on the classical metabolic enzyme ARG1 during the macrophage polarization process. Their investigations revealed a synchronous chronology between the escalation of ARG1 expression and the elevation of Kla levels, both of which exhibited a temporal lag of approximately 12 hours following the expression of nitric oxide synthase (NOS) 9. This temporal alignment suggests that Kla's existence is intertwined with post-inflammation repair processes. Eager to probe further, the researchers meticulously scrutinized over a thousand genes. Impressively, their findings showcased that the genes associated with damage-repair functions exhibited Kla modifications—a robust corroboration of their hypothesis 9. Subsequent experiments bolstered these findings, revealing the induction of Kla in M2-type macrophages. This revelation potentially underpins the aggregation of M2 cells within the TME 9.

**Figure 4:**
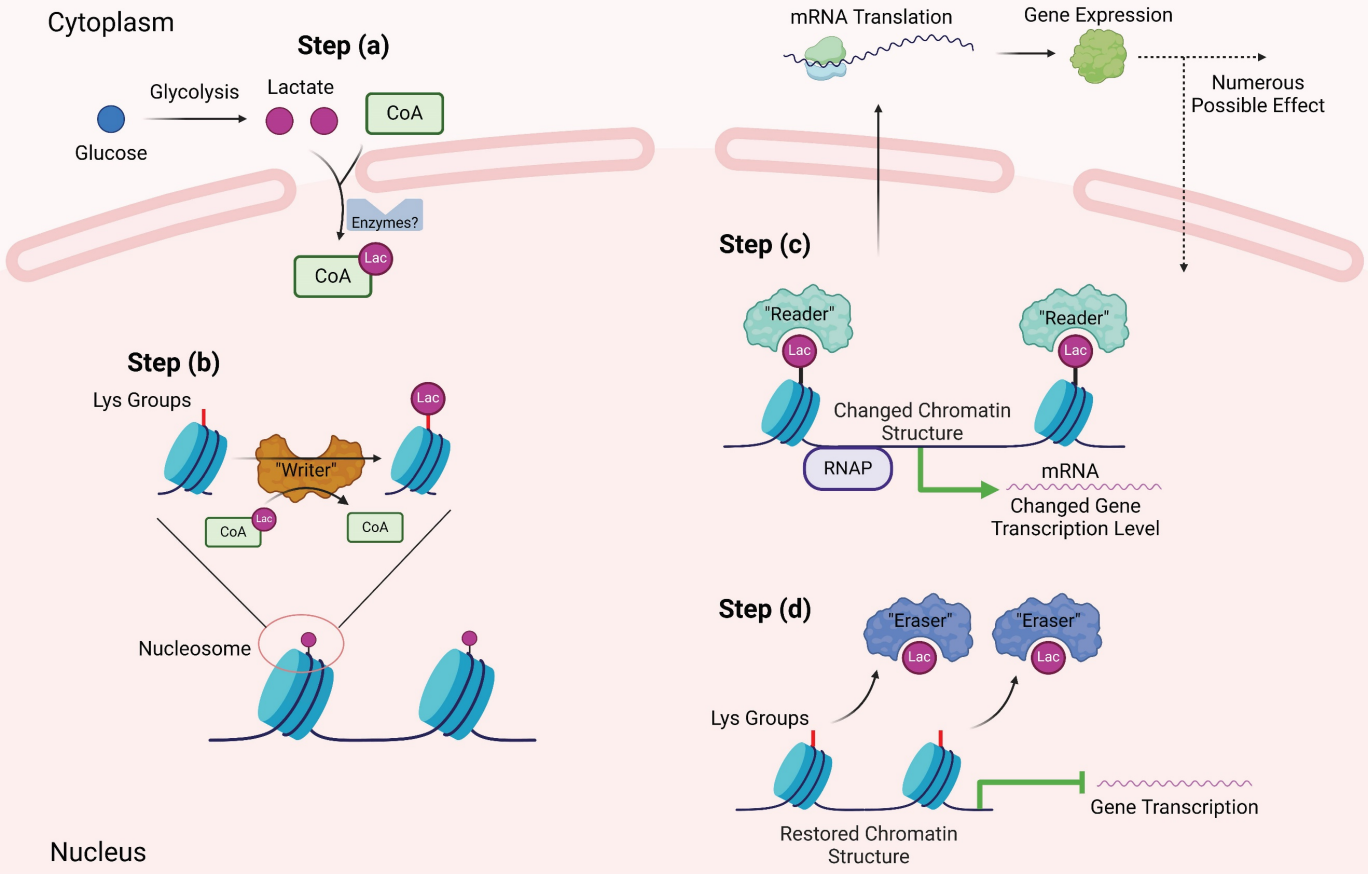
An ideal scheme of histone lactylation. Referring to other histone modifications, the process of histone lactylation might be as follows. Firstly, lactate from multiple pathways condenses with intracellular CoA to produce lac-CoA. Subsequently, lac-CoA enters the nucleus and, under the action of acyltransferase, known as "Writer," transfers the lactate acyl group to the lysine site. The chromatin structure will change after undergoing modification, and with the so called "Reader", the information contained in the structural changes will be identified, leading to changes in downstream gene transcription levels and further to variations in gene expression, protein function, and cell phenotype. Finally, after the modification is completed, "Eraser" recognizes the lactate acyl group and unbinds it from the lysine site, restoring the chromatin structure to normal. The restored lysine sites can be used for other HPTMs. At this point, the complete lactylation modification is thoroughly completed (Created with BioRender.com).

The research by Zhang et al. serves as foundation for further research. As anticipated, the quest for a deeper understanding of histone lactylation has led to inquiries concerning the involved intricate mechanisms. Foremost among these inquiries is the fundamental question of how cells engage lactate molecules in binding with lysine groups. Analogous to the elucidation of other HPTMs, one can suppose the comprehensive process of histone lactylation involves: a) the amalgamation of lactate and CoA to generate lac-CoA; b) the interaction of lac-CoA with the lactyl-acyl group on lysine residues, orchestrated by acyltransferase—an enzyme conventionally known as a "writer"; c) cells, facilitated by specific molecules known as "readers," adeptly interpreting the information conveyed by Kla and transmitting the information to affect gene expression; and d) ultimately, when this modification ceases to be requisite, certain molecules catalyze the erasure of the lactyl-acyl group; these entities assume the role of "erasers" (Figure 4). As of the present, substantial progress has been made concerning the three sequential phases, barring the lingering absence of identified "readers" within the domain of Kla. In the initial phase, scholars have yet to ascertain the existence of dedicated synthases that catalyze lac-CoA synthesis. However, a previous investigation has indicated that even in the absence of such enzymes, the collaboration between lactate and CoA persists, guided by the catalytic influence of the glyoxalase family. This orchestration employs reduced glutathione as an intermediary mediator, culminating in the eventual production of lac-CoA 68. In the subsequent phase, Zhang's work delineated the involvement of a prototypical protein, p300, previously recognized as a lysine acetyltransferase and now established as a “writer” for Kla. In this process, similar to many other HPTMs, the local structure of histones changes after lactylation, the "hairpin" of DNA becomes loose, and subsequently, the transcriptional activity of related genes is affected, resulting in diverse downstream effects. These assertions were further validated through analogous experiments conducted by other researchers, which yielded congruent outcomes 9, 69. Regarding the fourth sequential stage, a recent report revealed that SIRT3 can act as an "eraser" of Kla at the H4K16 site 70, which can also prevent the proliferation of cancer cells by reducing the activity of cyclin E2, a famous proliferation-related molecule 71. A study pertaining to Kla provides promising insights. Notably, class 1 histone deacetylases (HDAC1-3) play pivotal roles in diminishing Kla levels across diverse cell lines 72. However, another study provided a different explanation for the role of HDAC in the process of Kla. Lactylation at the H3K18 site is necessary for the progression of liver fibrosis, and HDAC seems to promote Kla expression. Pharmacological inhibition of HDAC decreases the lactylation level of H3K18 73. These two diametrically opposed conclusions lead to an interesting question: what is the spatiotemporal relationship between histone lactylation and other HPTMs? In fact, during the whole process of Kla modification, many molecules, including p300 and HDAC, can also functionally participate in other HPTM processes, especially acetylation, which is the most common and well-studied type of HPTM. In addition, the sites where they occur also have high similarity. Based on the findings of these two articles, the authors believe that there may be more than two ways of interaction between them. When these two agents are antagonistic, the two HPTMs occurring at the same site compete for inhibition. For example, in the process of liver fibrosis, high-level acetylation is a static feature of hepatic stellate cells (HSCs), and high levels of Kla indicate that HSCs initiate fibrosis progression 73. On the other hand, if they have similar biological roles, related enzymes, such as HDAC, can play a versatile role 72. If we think further, we will find that there must be a regulatory mechanism that can control the relationship between acetylation and lactylation, which is a fantastic scientific question that needs to be confirmed in future research.

Notably, the intrigue surrounding histone lactylation intensifies as it establishes numerous correlations with physiological and pathophysiological processes. This finding suggests that the modification of lysine sites transcends laboratory settings and holds tangible potential for application within medical practice. Recent investigations have described the implications of Kla for gene expression in normal tissues. Galle and colleagues documented the widespread occurrence of lactylation at the H3K18 site in both murine and human cells. These findings underscore that these modifications serve as distinctive hallmarks of tissue-specific enhancers 74. Histone lactylation at the H3K18 site has additionally been demonstrated to be associated with osteoblast differentiation, as it downregulates the expression of JunB, a well-known transcription factor. This cascade ultimately impacts the course of osteoblast differentiation 75, 76. Moreover, investigations have revealed that the induction of histone lactylation at pluripotency loci, facilitated by Glis1, effectively promotes the reprogramming of somatic cells 77. Furthermore, an ongoing clinical trial reported that lactylation at the H3K18 site can be used as an effective diagnostic indicator of septic shock 78. Another report noted that histone lactylation plays a role in the process of myocardial infarction and may be a promising therapeutic target 79. Histone lactylation has also been studied in parasitic infection 80. As an illustration, an extensive endeavor encompassing the identification of histone lactylation within malaria parasites and host cells has been undertaken. This undertaking revealed two novel lysine lactylation sites. Remarkably, the degree of lactylation at these sites is positively correlated with hyperlactemia, with the impacted genes being primarily associated with cellular invasion and the immune clearance mechanism of the spleen 81. Another pertinent example pertains to *Toxoplasma gondii*. Following a similar investigative approach, researchers have investigated lactylation sites within *Toxoplasma gondii*. This was followed by an encompassing bioinformatics analysis that consisted of Gene Ontology (GO) analysis, Kyoto Encyclopedia of Genes and Genomes (KEGG) analysis, and gene set enrichment analysis (GSEA). Notably, the outcomes revealed a distinctive pattern; the key enzymes of the glycolytic pathway in *Toxoplasma gondii* cells were disproportionately influenced by histone lactylation, albeit to varying degrees 82. In the context of Alzheimer's disease (AD), an important public health concern, there is a reported connection between such modifications and disease progression. An insightful study detailed the sequencing findings from Alzheimer's disease patients, revealing remarkably elevated levels of lactylation at H4K12 sites in microglia. These alterations directly precipitate an escalation in the downstream glycolytic pathways, prominently culminating in augmented expression of the pyruvate kinase PK-M2. This convergence establishes consequential positive feedback loop—that is, a synergy between glycolysis, lactylation at the H4K12 site, and heightened PKM2 activity 83. Lactylation at the H3K18 site can also promote the occurrence of AD and aggravate cognitive impairment through a similar mechanism 84. Of paramount importance is cancer, given its escalating threat to human well-being. The inherent intertwining of tumors and lactate metabolism signifies that histone lactylation will substantially propel the trajectory of scientific advancement within oncology, yielding pertinent outcomes—an avenue the authors discuss in the ensuing sections.

### 3.2 From histone lactylation to frontiers of oncology

In the preceding section, we have provided a comprehensive summary of the intricacies surrounding lactate metabolism and the ensuing modifications to histones. Subsequently, capitalizing on the importance of this paradigm-shifting discovery necessitates a strategic fusion of this knowledge with clinical practice. The inextricable interplay between cancer and lactate metabolism substantiates the relationship between histone lactylation and cancer development. Compared to various other HPTM modalities, lactylation is unique in its close connection with glucose metabolism. Notably, Zhang's research highlighted its influence on macrophages and hypoxic cells in their initial publication 9. Consequently, certain scholars have embarked upon discussions regarding the potential ramifications of this breakthrough within the domain of oncology research 85. Recently, a bioinformatics analysis was used to evaluate the relationships between histone lactylation and patient prognosis, immune cell infiltration, tumor cell invasion and proliferation in gastric cancer patients 86. With the continuum of research endeavors, a prevailing consensus has been solidified; histone lactylation plays a pivotal role throughout every stage of tumor development 23.

In this context, the authors have stratified these research findings into two principal categories, specifically delineating the impact of histone lactylation on both tumor cells and non-tumor cells—components integral to the TME. Among the many HPTM modalities, why is lactylation so closely related to tumors? This is a complex issue. The authors believe that the particularity of lactate molecules gives us the answer. The Warburg effect in tumor cells makes lactate a "star molecule" that cannot be overlooked. Moreover, lactate is widely distributed. It is generated mainly by glycolysis, which is an extremely conserved metabolic process that occurs in almost all cells, whether epithelial cells, immune cells or cancer cells. Finally, its important role is being discovered. Although it has long been considered metabolic waste, an increasing number of studies have confirmed the inaccuracy of this previous view. The effects of lactate on immune remodeling, metabolic reprogramming, and signal transduction are increasingly recognized by scholars. In the future, additional physiological and pathological phenomena related to lactylation may be recognized by researchers. Since the discovery of histone lactylation in 2019, related fields have gained considerable momentum, and another report linking histone lactylation with melanoma represents a new milestone 87. This report marks the pioneering exploration of the precise role of histone lactylation in distinct solid tumors, substantiating the previously hypothesized association between this form of HPTM and tumorigenesis. Consequently, this groundbreaking research has ignited a surge in related investigations, yielding a multitude of important findings (Table 2).

#### 3.2.1 Histone lactylation and non-tumor TME components

Zhang's discovery revealed the effect of lactylation on the polarization of macrophages 9. Throughout the intricate process of macrophage polarization, the genetic landscape, particularly that of genes associated with metabolic enzymes such as ARG1, undergoes pronounced modifications. These modifications, in turn, directly steer the polarization trajectory of macrophages toward the M2 phenotype. Importantly, these findings are consistent with corroborative research endeavors 88. This is a good start at connecting the mechanism of histone lactylation to the TME.

The role of macrophages in the TME is currently a hot topic for oncologic studies. A recent study published earlier this year has shed light on the intricate relationship between macrophage polarization and histone lactylation. In this investigation, researchers revealed a noteworthy finding: the polarization of TAMs and the concurrent histone lactylation within these cells do not hinge on intrinsic glycolytic metabolism. Another study illustrated a deeper link between histone lactylation and macrophage metabolism. In M2 macrophages, mitochondria are highly fragmented, and this phenomenon is closely related to the special metabolic changes in M2 cells 89. The histones inside fragmented M2 cells undergo pan-lactylation, which results in positive feedback and drives macrophages to continuously polarize toward M2, thereby affecting the TME 89. However, even when extracellular lactate is transported into the cellular interior, it retains the capacity to incite lactylation 90. In the TME, lactate mainly enters macrophages through the MCT1 molecule, and these lactate molecules are involved in two downstream pathways. First, they can enter mitochondria to provide energy in a process mediated by the mitochondrial pyruvate carrier (MPC). Another option is independent of MPC, whose main role is to promote HIF-1α stability 90. Inside macrophages, the second pathway mentioned above is one of the main driving forces driving macrophage polarization through lactylation of the H3K18 site 90. Similar studies have shown that lactylation of the crucial lactate metabolic enzyme PK-M2 facilitates the trajectory of macrophage differentiation toward the phenotype associated with injury repair and proinflammatory responses, which, together with the findings of previous reports, suggest that the flow of lactate drives macrophage polarization 91. Other studies have provided an alternative perspective to elucidate the phenomenon of macrophage transformation. In the context of an inflammatory milieu, macrophages encounter pathogen-associated molecular patterns (PAMPs) or damage-associated molecular patterns (DAMPs), which are discerned by cell-surface recognition receptors. In response, these receptors initiate dual actions. First, they induce the production of molecules such as tumor necrosis factor (TNF), IL-6, and IL-22, eliciting responses to the inflammatory setting. Simultaneously, these molecules affect neighboring cells through the activation of the NF-κB and AP-1 signaling pathways 92, 93. Second, they can also activate glycogen synthase kinase 3β and fork head box protein O1 through the PI3K-Akt pathway, thus regulating inflammatory responses 94-98. Previously, researchers have pinpointed the B-cell adaptor for PI3K as a pivotal regulator intimately linked to toll-like receptor (TLR) signaling. This molecule serves as a critical juncture in modulating the downregulation of inflammatory responses within macrophages 99-101. Further investigation revealed that BCAP significantly upregulated the expression of genes related to the suppression of the inflammatory response by histone lactylation, which drives macrophages to inhibit inflammation and promote tumor progression 102. Another study confirmed the above findings from another perspective. By reducing the level of histone lactylation in macrophages in a variety of ways, macrophages can restore endocytosis and antitumor immune function to some extent 103.

Myeloid-derived cells are also an important part of the TME. They can migrate to the tumor site through a variety of ways and contribute to the immunosuppressive microenvironment. In a recent investigation, while not explicitly categorized under the domain of oncology, a study provided additional information on the intricate mechanism and functional importance of lactylation in BMDMs, thereby expanding our perspective 104. Notably, numerous earlier studies have consistently demonstrated marked elevations in lactate and HMGB1 levels during sepsis 105-107. Building upon this foundation, researchers have shown that lactate is the main driving force of HMGB1 accumulation in BMDMs 104. This pathway involves the elevation of lactate levels, which triggers the lactylation of specific sites on histones, consequently influencing the expression of HMGB1. However, researchers have made even more remarkable findings; heightened lactate levels can stimulate the acetylation of other lysine sites. This process is facilitated through the inhibition of SIRT1, a recognized "eraser" responsible for acetylation 108. Subsequently, researchers investigated murine BMDMs further, noting that the phosphorylation and subsequent degradation of YAP and LATS1, which are upstream molecules of SIRT1, are key steps in the lactate-driven process 104. This study underscores the intricate interplay between various HPTMs. One can envision a complex network of histone modifications across diverse cell types, tissues, and diseases, yielding innumerable impactful effects awaiting exploration. Such HPTMs are not exclusively present in macrophages; in tumor-infiltrating myeloid cells, amazing discoveries have also been reported. Xiong and colleagues reported the lactylation of the H3K18 site, revealing a robust correlation with increased METTL3 expression. METTL3, an RNA binding protein, orchestrates m^6^A RNA modifications. Further exploration revealed that METTL3 overexpression in myeloid cells directly influenced Jak1 pathway activity. This assertion was substantiated by Western blot and bioinformatics analyses 109. Notably, altered Jak1 dynamics have consequential impacts on downstream STAT3, a widely recognized oncogenic regulatory signaling conduit 110, 111. Increased STAT3 activity induces the production of an array of tumor-promoting molecules, including IL-6, IL-10, and iNOS 109.

Innate immune cells are an integral part of the human immune system. In a state of tumor burden, cells from the innate immune system are also affected by the TME and have complex interactions. The initial documentation of histone lactylation in innate immune cells emerged from Wang and his collaborators' work, who focused on Natural Killer T (NKT)-like cells in malignant pleural fluid, which is often characterized as an immunologically inert zone within the body 112. Most NKT-like cells in the malignant pleural fluid have a CD4^+^FOXP3^+^ phenotype, and cells with this phenotype often express immunosuppressive cytokines, including CTLA-4, IL-4, and transforming growth factor (TGF)-β 113. The investigation revealed that within the lactate-rich environment of malignant pleural fluid, the FOXP3 promoter site within FOXP3^+^ NKT-like cells exhibited elevated levels of histone lactylation, which were notably attenuated by the specific lactate transporter inhibitor 7ACC2. This observation revealed the role of histone lactylation as a negative immunoregulator in NKT-like cells, ushering in novel concepts and potential therapeutic targets for clinical intervention 112.

T cells are a large immune cell population with complex functions in tumors, and the role of T cells in cancer is also an absolute hot topic in the field of oncology. There are many studies on the relationship between histone lactylation and T cells. For instance, in Th17 cells, lactylation at the H3K18 site can facilitate the transformation of inflammatory T cells to regulatory T cells, consistent with the findings of the aforementioned studies 114. Another study focused on T-cell responses in acute myeloid leukemia (AML). In AML cells, high expression of STAT5 can increase the transcription of a variety of glycolytic enzymes. Subsequently, lactylation occurs at H3K18, H4K5, H4K8 and H4K12 115. Changes in H4K5 sites significantly increase the expression of PD-L1, which disables most T cells but also increases the efficacy of immune checkpoint inhibition (ICI) therapy to a certain extent 115. The same results have been reported in prostate cancer 116. The inhibitory effect of histone lactylation on T-cell activity has also been found in glioma. Lactate can promote the expression of CD39 and CD73 in a variety of cells in the tumor microenvironment, which are the main characteristics of the inhibitory TME. Finally, this leads to the exhaustion of CD8^+^ T cells and an increase in Treg cells 117.

Finally, a crucial constituent of the tumor microenvironment that cannot be overlooked is the intricate network of microvessels. While a comprehensive and thorough investigation unraveling the precise interplay between histone lactylation and tumor microvascular formation is currently lacking, a study concentrating on retinal microvascular formation has expanded our comprehension. Wang et al. reported that elevated intracellular lactate levels in microglia can stimulate angiogenesis. Subsequent bioinformatics data analysis revealed the YY1 gene as a crucial factor in lactate-driven angiogenesis. Substantiating their findings, experimental data indicated that p300, a 'Writer' enzyme for lactylation, facilitates histone lactylation to augment YY1 expression, consequently promoting angiogenesis via the downstream fibroblast growth factor (FGF) pathway 118. Another study on prostate cancer also confirmed the promoting effect of lactylation on tumor angiogenesis 116. Given the profusion of angiogenic factors and elevated lactate concentrations within the TME, a pertinent inquiry arises: could analogous mechanisms be operative in the TME? Might this lead to novel therapeutic strategies for cancer? These questions require further investigation.

Both histone lactylation and the TME represent burgeoning avenues of research, as there are a limited number of current publications. Moreover, the literature has yet to thoroughly integrate these two areas. However, forthcoming investigations and reports will undoubtedly emerge, offering deeper insights into the intricate interplay between histone lactylation and the TME. These anticipated developments are poised to reveal additional mysteries in this evolving field.

#### 3.2.2 Histone lactylation and tumor cells

It is well known that tumorigenesis arises from the dysregulation of cell division 119. Histone lactylation constitutes a crucial form of epigenetic regulation. Therefore, these modifications may play a role in the initiation of tumorigenesis 120. The epithelial mesenchymal transition (EMT) is a pivotal stage in both the initiation and metastasis of cancer. Recent attention has been given to a study involving lactylation and EMT in the context of myocardial infarction. Despite the nontumorous focus of this study, it offers novel insights into the interplay between tumor origin, cancer metastasis, and histone lactylation. In MI tissue, hypoxia leads to a notable increase in glycolytic metabolism and subsequently increased lactate levels. Within this context, researchers have observed an elevation in histone lactylation levels, directly fostering the expression of Snail1—a cis transcription factor—thereby promoting EMT through the TGF-β/SMAD2 axis 121. A novel hypothesis is that analogous metabolic disturbances and epigenetic modifications might also transpire during the process of tumor initiation, revealing potential novel mechanisms and therapeutic targets. One study confirmed that reduced lactylation at H3K9 and H3K14 sites could stop the origin of hepatocellular carcinoma (HCC) 122. Another investigation is about liver fibrosis, which is an important stage in the development of HCC 123. When liver fibrosis is initiated in HSCs, the expression of a series of enzymes related to digestion, represented by hexokinase 2 (HK2), increases, resulting in the massive production of lactate. Subsequently, lysine residues at H3K9, H3K18, H4K8 and H4K12 were lactylated, further affecting the expression of glucose metabolism genes and accelerating the process of liver fibrosis 73. Further studies also showed that lactylation occurring inside HSCs is HDAC-related. When HDAC is inhibited by inhibitors, the lactylation level of H3K18 decreases, and the acetylation level increases 73. This shows that the above assumption is reasonable to some extent. Certain researchers have employed gastric cancer cells as a representative sample to gauge the extent of histone lactylation. Their findings revealed that genes possessing regulatory functions, particularly those associated with DNA modification and RNA processing enzymes, exhibited elevated levels of lactylation 124. Several studies have shown that in multiple kinds of tumor cells, higher lactate and LDH levels are strongly correlated with poor patient outcome, and these levels are dependent on the lactate-metabolizing activity of tumor cells; moreover, the treatment effect can be improved by pretreatment with lactate. The above results can be proven by both experimental data and meta-analysis 125-127. The investigation and screening of the relevant genes in the above experimental findings eventually revealed that the expression of YTHDF2, a 'reader' protein of RNA methylation, exhibited a clear positive correlation with histone lactylation levels 87. Simultaneously, researchers have observed substantial downregulation of the TP53 and PER1 genes, both of which are recognized tumor suppressor genes, in tumor cells. The investigators proposed that this downregulation could also be linked to the elevated expression of YTHDF2 and increased lactylation levels 87. Other distinct forms of tumor origin associated with histone lactylation have also been reported. Wang et al. demonstrated that enteropathogenic bacteria may promote tumorigenesis by affecting histone modifications in normal cells 128. They found that during the infection process into the gut of *S. typhimurium*, normal cells produce LINC00152, a long non-coding RNA (lncRNA) that has been shown to have a clear oncogenic role in colorectal cancer (CRC) 129, 130. This lncRNA can in fact promote tumorigenesis by influencing histone lactylation to prevent mitotic control 128.

Immune escape is an important feature of tumors. Many studies have reported the relationship between histone lactylation and immune recognition dysfunction. Objectively speaking, histone lactylation plays a role in promoting tumor cell immune escape. A recent publication in the current year centered its investigation on lactylation rather than focusing solely on general blood lactate and LDH levels. The research included disease-related data sourced from more than 800 patients (with 300 sourced from the TCGA database) via various public datasets. The researchers commenced by conducting a differential expression analysis through which they identified six genes associated with lactylation. Subsequent univariate survival analysis revealed that, in addition to NUP50 and EFNA3, which exhibited some protective tendencies, the remaining four genes were negatively correlated with patient survival and prognosis. To further explore this phenomenon, the researchers categorized patients into two groups based on their lactylation levels (high and low) and constructed a predictive model. As anticipated, elevated lactylation levels were found to significantly impact patient prognosis. Moreover, the GSEA and KEGG analyses demonstrated substantial enrichment of relevant genes involved in processes such as cell proliferation, angiogenesis, and the TGF-β signaling pathway. This concurs with observations of a diminished immune response and the emergence of tumor immune evasion 86, 131. In AML, histone lactylation increases the expression of PD-L1, which increases the likelihood of immune escape from tumor cells 115.

An important feature of tumor progression is the increase in the absolute number of tumor cells, which is closely related to the uncontrolled proliferation of tumors. An intricate positive feedback loop instigated by the interplay between histone lactylation and accelerated tumor proliferation within renal cell carcinoma (RCC) tissues has been reported, with a crucial connection to the Von Hippel Lindau (VHL) gene 132. The VHL gene is positioned on the 3q25-26 region of the human chromosome and is a well-established tumor suppressor gene. It encodes a 213-amino-acid VHL protein that effectively triggers the ubiquitin degradation pathway, leading to the degradation of HIF and consequently curbing the proliferation of cancer cells 133. Within RCC cells, the accumulation of lactate initiates intracellular histone lactylation, concurrently suppressing the expression of the VHL gene. As a consequence, the degradation of HIF is decreased, permitting increased reception of growth signals by the tumor. This, in turn, fosters rapid tumor proliferation, yielding heightened lactate production within the TME and, consequently, culminates in elevated glycolytic metabolic activity and the enhanced expression of pertinent genes in tumor cells. Collectively, these processes delineate a comprehensive positive feedback pathway that serves to fuel tumor proliferation 132. In addition, increased levels of histone lactylation in RCC also result in increased platelet-derived growth factor receptor β (PDGFR-β), whose increase is an important factor in promoting tumor proliferation 132. Diapause refers to the phenomenon in which cancer cells pause or reduce their proliferation rate under a variety of stress conditions 134. In CRC, SMC4 was discovered as a new diapause-related gene 135. In the state of diapause, the metabolic rate of cells does not necessarily decrease as much as the rate of proliferation. In fact, in SMC-related diapause CRC cells, the expression of multiple rate-limiting enzymes involved in glycolysis increases, and histone lactylation at H4K8, H4K12, and H3K14 increases accordingly 135. Investigators subsequently found that lactylation at the H4K12 site is strongly related to the ABC transporter system and promotes drug resistance in diapause CRC cells through drug efflux 135. Another study on CRC confirmed that lactylation at the H3K18 site can activate the RUBCNL gene at the transcriptional level, a promoter of autophagy, leading to rapid proliferation and drug resistance in CRC cells 136. Finally, a study on tumor stem cells confirmed the promoting effect of histone lactylation on tumor proliferation. Metabolic reprogramming of glioma cells increases the level of H3K18 site in lactating cells, resulting in the activation of transcription of the downstream gene LINC01127. LINC01127 can not only activate the JNK pathway by CIS regulating MAP4K4 but also directly activate the NF-κB pathway, which leads to the rapid self-renewal of glioma stem cells, which is characterized by high-level expression of a series of molecules, including Nestin and CD44 137.

Another tumor-promoting consequence of this modification arises from its influence on genes crucial to tumor metabolism because energy is the basis of all biological behaviors. Lactate is inextricably linked with tumor energy metabolism. Therefore, the close correlation between lactylation and tumor proliferation is a reasonable scientific hypothesis. Fortunately, this idea has been supported by a large amount of experimental data. For instance, within non-small cell lung cancer (NSCLC), a number of genes intricately connected to glycolytic metabolism, including HK-1, G6PD, PKM, SDH, IDH, and HIF-1a, exhibit noteworthy elevations in histone lactylation levels 138. In lung cancer, metabolic reprogramming resulting from an increase in IDH3G at the core can also increase the lactylation level of H3K18 139. Correspondingly, LDHA-mediated metabolic reprogramming in gastric cancer can also affect the lactylation of H3K9, H3K18, and H3K56 140. Pan et al. recently reported a significant association between high lactylation at both H3K9 and H3K56 and the progression of HCC. Their research also led to the identification of a potential therapeutic agent named demethylzeylasteral (DML), which effectively inhibited cell division, restrained tumor cell migration, and induced cell apoptosis in HCC cells. Further bioinformatics analysis demonstrated that genes targeted by DML were enriched in the glycolysis pathway. Moreover, this study explored the complete relationship between the DML/glycolysis/histone-lactylation axis and HCC stem cells, thereby providing insights into the contributory role of histone lactylation in the progression of HCC 141. In another study, hepatitis B virus-associated HCC was the subject of investigation. The researchers meticulously identified 9275 potential lactylation sites within the proteome of liver cancer cells, 19 of which were found on histones. Remarkably, the downstream genes associated with these loci covered an extensive spectrum of cellular metabolic pathways spanning the tricarboxylic acid cycle, amino acids, fatty acids, and nucleotide metabolism. This intricate regulation of cellular metabolism through lactylation has been shown to promote tumor progression 142. Recently, researchers found a circular RNA named CircXRN2 in the sequencing results of bladder cancer and prostate cancer tissues. Its main role is to activate the Hippo pathway, a well-known tumor suppressor pathway, by stabilizing LATS1 molecules 143. Subsequently, the anaerobic metabolism in tumor cells decreases, the lactylation of H3K18 sites decreases, and the multiple malignant biological behaviors of tumors also diminish 143. In head and neck squamous cell carcinoma (HNSCC) cells, elevated levels of histone lactylation mark a highly aggressive subpopulation of cells within the tumor tissue, and there is a distinct differential expression of genes related to glycolysis in this subpopulation, which may be associated with histone lactylation, although the evidence is not clear 144.

Given the capacity of histone lactylation to govern genes, it is reasonable to deduce that this regulatory function should not be confined solely to metabolic pathways, as genes, despite their diversity, share similarities in structure and composition. Therefore, histone lactylation must affect tumor progression in a variety of ways. This supposition has garnered empirical validation. Alterations in the expression of oncogenes, tumor suppressor genes, transcription factor genes, and genes associated with cell proliferation have been observed in breast cancer cells. Notably, a robust correlation has been established between these changes in expression and lactate levels 145. A second study, which has greater representational value, elucidated the regulatory capacity of histone lactylation on non-glycolytic metabolic genes. Research findings have indicated that histone lactylation plays a role in promoting the expression of neuroendocrine genes in lung and prostate cancer cells 146. These alterations in gene expression have been linked to the presence of Numb, a determinant of cell fate that typically associates with Parkin to regulate the processes of cell division and differentiation 147-149. Given the widespread cooperation of the glycolytic metabolic pathway by tumor cells and their subsequent mitochondrial dysfunction, an environment conducive to heightened histone lactylation levels has been created, and these findings were corroborated by multiple earlier studies. This mitochondrial dysregulation can also impact the Numb/Parkin pathway. The perturbation of cellular metabolism, coupled with the escalated expression of neuroendocrine genes, provides a comprehensive explanation for the transformation of lung and prostate cancer cell phenotypes into those resembling neuroendocrine tumors. This transformation, on a macroscopic scale, often results in diminished efficacy of chemotherapy and targeted agents 146.

**Figure 5:**
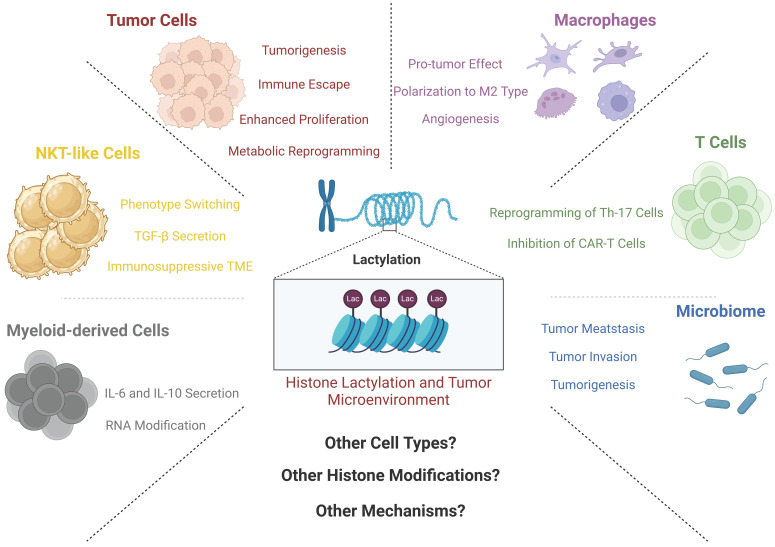
Overview of effects of histone lactylation and TME components. This figure shows main effects by which histone lactylation affects tumor cells and other TME components. All key cell types and downstream effects are marked with different colors. As is shown above, histone lactylation has various effects on cancers and other diseases (Created with BioRender.com).

Only four years have elapsed since the initial discovery and reporting of histone lactylation; thus, the limited volume of literature and the relatively shallow depth of related research are understandable (Figure 5). Nonetheless, it is imperative to acknowledge the immense potential of this research direction. Given the consistent occurrence of the Warburg effect, pervasive glycolytic metabolism in tumors, and abundant accumulation of lactate both within and outside tumor cells across diverse tumor types, the importance of lactate and its associated epigenetic modifications for tumor biology cannot be underestimated. As time progresses, a more comprehensive body of research is expected to emerge, leading to a deeper understanding of the specific role played by histone lactylation in the intricate progression of tumorigenesis.

## Perspective and conclusion

The identification of histone lactylation, coupled with the ongoing advancements in subsequent investigations, has provided insight into this newfound HPTM mechanism and associated functions. It involves the century-old cancer hallmark of conserved glycolytic metabolism, the Warburg effect and epigenetic regulation. A wide variety of cells, including tumor cells, can be affected broadly by lactylation. Investigations on lactate have led to new discoveries. However, it is imperative to acknowledge that our understanding of this topic is not comprehensive, and a considerable number of inquiries pertaining to histone lactylation have not been elucidated 150. The first concern pertains to an incomplete logical explanation. Although we have established a correlation between lactate accumulation and histone lactylation, the following questions still remain: does an increase in lactate always result in heightened lactylation? Additionally, we need to ascertain the specific histone sites that undergo lactylation. Thus, why these sites undergo lactylation while others do not should be investigated. Another issue arises from the classical HPTM mechanism. Other HPTM modifications, such as succinylation and palmitoylation, involve potential sites other than lysine. This raises the following question: are there other amino acids targeted for lactylation 151? Is there any crosstalk between histones and other HPTMs? The final question, which has been widely raised, pertains to the identification of reader proteins involved in the histone lactylation process. Moreover, further investigations have explored whether there are additional writer and eraser enzymes other than p300 and HDACs. Additionally, there is a need to determine whether the enzyme responsible for catalyzing lac-CoA production is involved. These inquiries necessitate further exploration by researchers and represent the future direction of related scientific investigations. With the passage of time and increased research efforts, these questions are likely to be definitively addressed. Despite the existence of numerous unknowns, it is important to acknowledge the potential contribution of this mechanism to future advancements in the field of human medicine.

As histone lactylation is prevalent in various cell types, therapies targeting this process have the potential to exert a broad range of effects, even though limited studies are currently available. One potential effect pertains to the remodeling of tumor cell metabolism. Given that histone lactylation significantly influences the expression of metabolism-related genes in tumor cells, as previously discussed, specific interventions to block the lactylation modification process could partially reverse the shift of tumor cells toward glycolysis. Another avenue for exploration involves the regulatory influence of lactylation on the TME. Notably, this process includes the modulation of macrophage polarization, with the aim of steering a greater number of macrophages toward the M1 phenotype, thereby counteracting the inhibitory effect of the TME 9. Moreover, considering the aforementioned connection between histone lactylation and NKT cells, targeted inhibition of this modification process has the potential to modulate T cell phenotypes 112. Hence, a promising outlook has emerged for clinical applications focused on targeting lactate modifications. The identification of treatment targets for various diseases, notably tumors, is particularly probable. A conceivable approach involves the design and synthesis of specialized compounds aimed at either degrading lactate within the tumor microenvironment or inhibiting lactate transport. Such interventions could result in the inhibition of lactylation and consequently decelerate tumor growth 152. This trajectory aligns with the precedent set in 2007, in which MCT1 was highlighted as a viable target for immunotherapy, marking the inception of a tangible therapeutic strategy centered on blocking intratumoral lactate transport 153. Current research advances have largely confirmed the role of blocking the lactate cycle in tumor therapy. When the transport of lactate is inhibited, lactate cannot enter the glycolysis pathway, which forces tumor cells to undergo a metabolic shift and acts to inhibit tumor angiogenesis 46, 154. Additionally, acidification of the TME can be mitigated, and issues related to both the incapacitation of immune cells and the suppressive immune microenvironment can be addressed 37. In cells undergoing glycolysis, the cellular cytosolic pH decreases, which leads to impaired tumor cell metabolism and loss of the ability to regulate the pH of the microenvironment and cytosol 155. Pharmaceutical agents developed based on the aforementioned mechanisms have progressed into clinical trials and effectively impeded lactate transport. Tumor tissue lacking MCT1 displays subdued metastatic activity, thereby providing additional corroboration for the feasibility of targeted lactate transport therapy from an alternative vantage point 156. Another possibility worth exploring involves the design of inhibitory agents targeting pivotal nodes of histone lactylation, thus enabling potential precision interventions. Notably, drugs inhibiting other HPTMs have already been incorporated into clinical practice and demonstrated meaningful efficacy 157. A more feasible strategy could involve the synergistic integration of histone lactylation inhibitors and immunotherapeutic agents aimed at the TME, resulting in enhanced and targeted tumor suppression. In the context of emerging nanotechnology, the utilization of nanoparticles as carriers for drug delivery has potential for concurrently, precisely, and systematically transporting diverse drugs via distinct mechanisms to tumor tissues. In summary, both scientific and clinical advancements are needed to yield a more holistic and precise understanding of the histone lactylation mechanism involved. As we look ahead, promising breakthroughs within this realm are anticipated in future studies.

In summary, this review provides a comprehensive exploration utilizing lactate as an important molecule in processes spanning from tumor lactate metabolism to histone lactylation and further to downstream gene regulation and the development of cancer. Previously regarded as a mere metabolic waste byproduct, lactate has emerged as an important player intricately tied to tumor initiation and advancement. The scope of tumor lactate metabolism extends beyond glycolysis alone. It covers a multifaceted network encompassing lactate production, consumption, transportation and signal transduction. In a pivotal breakthrough in 2019, Zhang et al. revealed the prominent link between lactate and a new HPTM—histone lactylation. Meticulous elucidation of the discovery process has shed light on the role of histone lactylation during macrophage polarization 9. This seminal revelation engendered considerable intrigue and inaugurated an uncharted domain of investigation. Subsequently, intensive inquiry has been made into the mechanics of histone lactylation, encompassing the identification of additional lysine sites, the characterization of writers and erasers, and related aspects. The details of the intricate facets of histone lactylation are becoming increasingly clear. Concurrently, several researchers have endeavored to bridge the gap between this mechanism and associated pathologies, constituting another important facet of this review. Since its discovery, histone lactylation has been discerned across various cancer cell types, such as gastric, breast, prostate, and lung cancer cells. Furthermore, immune cell functions—macrophage and NKT-like cells—are influenced by histone lactylation. As implicated in diverse dimensions of tumor growth, histone lactylation impacts metabolism, fosters drug resistance, and exerts a regulatory influence on immune responses. This succinctly encapsulates the crux of the review, which provides novel perspectives among scientific researchers and clinicians alike. In the imminent future, the enigma of histone lactylation will be fully unraveled, paralleled by consequential clinical applications yielding affirmative outcomes. Ultimately, these endeavors will significantly contribute to human health, improving the lives of a broader spectrum of patients.

## Figures and Tables

**Table 1:** Relations between lactate and immune TME components

Components	Mechanisms	Effect	References
Macrophages	Reduced NF-κB activation, cytotoxic cytokines secretion, cell migration; Enhanced ERK-STAT3 pathway, GPR132 and Notch pathway; HIF-1a stabilization; Histone lactylation	M2 polarization; Pro-tumor effect	9, 64, 158-162
CD4+ T cells	Reduced cell motility; Inhibited TCR triggering; Enhanced PD1/PD-L1 pathway, fatty acid synthesis; Acidic pH TME	⬇ CTL function ⬇ anti-tumor immunity ⬇ T cell proliferation ⬆ Secretion of IL-17	163-168
CD8+ T cells	Reduced NAD availability, lactate efflux, cell motility, JAK-JUN pathway, cytotoxic cytokine production; Enhanced PD-1/PD-L1 pathway; Acidic pH TME	⬇ CTL function ⬇ Anti-tumor immunity ⬇ T cell proliferation ⬆ Apoptosis	163, 169-172
Dendric cells	Enhanced GPR81 signaling; Reduced TLR signaling	⬆ Secretion of IL-10 ⬇ Secretion of IL12, IL-6 and IFN-γ	58, 62, 173-177
Myeloid Derived Suppressor Cells	Enhanced cell secretion	⬆ Secretion of G-CSF and GM-CSF	178-180
Regulatory T cells	FOXP3-mediated repression of Myc	⬆ Tregs differentiation and proliferation	181-183
Natural Killer cells	Reduced NFAT activity, NKp46 activity, mTOR signaling pathway; Acidic pH TME	⬇ Cytotoxic function ⬇ Secretion of IFN-γ ⬆ Secretion of TGF-β and IL-10 ⬆ Apoptosis	184-189

**Table 2:** An overview of relations between histone lactylation and clinical investigations

Disease Type	Cell Type	Lactylation Site	Related Molecules or Pathways	Downstream Effect	References
Alzheimer's Disease	Microglia	H4K12, H3K18	PK-M2	Cognitive impairment	83, 84
Normal Tissue	Murine and human cells	H3K18	JunB, Glis1	Cell differentiation and reprogramming	74-77
Septic Shock	Not mentioned	H3K18	Not mentioned	Not mentioned	78
Not mentioned	Macrophages	Pan-lactylation	ATF4/c-Jun pathway, ARG1	Polarization to M2 type	89
Lung Carcinoma	Macrophages	H3K18	ARG1	Polarization to M2 type	90
Inflammatory Bowel Disease	Macrophages	Pan-lactylation	BCAP	Polarization to M2 type	100
Prostate Cancer	Macrophages	H3K18	Wnt/β-catenin pathway	Promotion of tumor growth	103
Colorectal Cancer	Myeloid Cells	H3K18	METTL3	Secretion of IL-6 and IL-10; Promotion of immunosuppressive TME	109
Malignant Pleural Effusion	NKT-like Cells	H3K18	FOXP3	Promotion of immunosuppressive TME	112
Not mentioned	Th-17 Cells	H3K18	FOXP3, IL-17	Promotion of reprogramming of Th-17 cells	114
Acute Myeloid Leukemia	Tumor Cells	H3K18, H4K5, H4K8 and H4K12	PD-L1	Reduction of T cell response; Promotion of immune escape	115
Prostate Cancer	Tumor Cells	H3K18	PD-L1	Promotion of angiogenesis and immune escape	116
Glioblastoma	CAR-T Cells	H3K18	Ectonucleotidase, CCR8	Reduction of CAR-T therapy	117
Retinal Diseases	Macrophages	Pan-lactylation	YY1	Promotion of angiogenesis	118
Myocardial Infarction	Epithelial Cells	Pan-lactylation	TGF-β/SMAD2 pathway	Promotion of endothelial-to-mesenchymal transition	121
Hepatocellular Carcinoma	Tumor Cells	H3K9, H3K14	Glycolytic related enzymes	Reduction of tumor lactate production	122
Liver Fibrosis	Hepatic Stellate Cells	H3K9, H3K18, H4K8, H4K12	Hexokinase-2	Activation of hepatic stellate cells	73
Ocular Melanoma	Tumor Cells	H3K18	YTHDF2, TP53 and PER1	Promotion of tumorigenesis	87
Colorectal Cancer	Tumor Cells, Microbiomes	H4K8, H4K5	LINC00152	Promotion of tumorigenesis	128
Gastric Cancer	Not mentioned	Not mentioned	Cuproptosis-related genes and lactylation-related genes	Reduction of immune response; Promotion of immune escape	86, 131
Renal Cell Carcinoma	Tumor Cells	H3K18	VHL, PDGFRβ	Promotion of tumor proliferation	132
Colorectal Cancer	Diapause-like Tumor Cells	H4K8, H4K12, H3K14	SMC4, ABC transporter	Promotion of drug resistance	135
Colorectal Cancer	Tumor Cells	H3K18	RUBCNL	Promotion of drug resistance	136
Glioblastoma	Tumor Stem Cells	H3K18	MAP4K4/JNK pathway, LINC01127, NF-κB pathway	Promotion of self-renewal of tumor stem cells	137
Lung Carcinoma	Tumor Cells	H3K18	BZW2	Metabolic reprogramming	139
Lung Carcinoma	Tumor Cells	H3K9, H3K18, H3K56	AKR1B10	Metabolic reprogramming; Promotion of drug resistance	140
Hepatocellular Carcinoma	Tumor Cells	H3K9, H3K56	Glycolytic-related enzymes	Metabolic reprogramming	141
HBV-Related Hepatocellular Carcinoma	Tumor Cells	Over 9000 lysine sites	Multiple metabolic pathways	Metabolic reprogramming	142
Bladder Cancer	Tumor Cells	H3K18	CircXRN2, LATS1, LCN2, Hippo pathway	Promotion of glycolysis	143
Lung Carcinoma and Prostate Cancer	Tumor Cells	H3K18	Numb/Parkin pathway	Neuroendocrine differentiation; Promotion of drug resistance	146

## Abbreviations

LDH: lactate dehydrogenase
TME: tumor microenvironment
HPTM: histone post-translational modification
OXPHOS: oxidative phosphorylation
TCA: tricarboxylic acid
ASCT2: type 2 amino-acid transporter
SNAT2: sodium-coupled neutral amino-acid transporter
α-KG: α-ketoglutaric acid
PDAC: pancreatic ductal adenocarcinoma
MCT: monocarboxylate transporters
SLC: solute carrier family
HIF: hypoxia inducible factor
GPCR: G-protein-coupled receptor
IFN: interferon
APC: antigen-presenting cell
TAM: tumor associated macrophage
IL: interleukin
VEGF: vascular endothelial growth factor
ARG1: arginase 1
BMDM: bone marrow-derived macrophage
Kla: lysine site lactylation
NOS: nitric oxide synthase
HSC: hepatic stellate cell
HDAC: histone deacetylases
GO: Gene Ontology
KEGG: Kyoto Encyclopedia of Genes and Genomes
GSEA: Gene Set Enrichment Analysis
AD: Alzheimer's Disease
PAMP: pathogen-associated molecular pattern
DAMP: damage-associated molecular pattern
TNF: tumor necrosis factor
TLR: toll-like receptor
NKT cell: natural killer T cell
TGF: transforming growth factor
AML: acute myelocytic leukemia
ICI: immune checkpoint inhibition
FGF: fibroblast growth factor
EMT: epithelial mesenchymal transition
HK2: hexokinase 2
lncRNA: long non coding RNA
CRC: colorectal cancer
RCC: renal cell carcinoma
VHL: Von Hippel Lindau
PDGFR-β: platelet-derived growth factor receptor β
NSCLC: non-small cell lung cancer
HCC: hepatocellular carcinoma
DML: demethylzeylasteral
HNSCC: head and neck squamous cell carcinoma
